# Induction of Labour among Pregnant Women in the Department of Obstetrics and Gynaecology in a Tertiary Care Centre

**DOI:** 10.31729/jnma.8255

**Published:** 2023-09-30

**Authors:** Siddhartha Kumar Yadav, Indra Yadav, Tarun Pradhan, Sabita Jyoti, Rozy Yadav

**Affiliations:** 1 Department of Obstetrics and Gynaecology, Birat Medical College Teaching Hospital, Biratnagar, Morang, Nepal; 2 Department of Community Medicine, Nepalgunj Medical College and Teaching Hospital, Kohalpur, Banke, Nepal; 3 Department of Obstetrics and Gynaecology, Karnali Province Hospital, Kalagaun, Surkhet, Nepal

**Keywords:** *induction of labour*, *misoprostol*, *pregnancy*

## Abstract

**Introduction:**

Induction of labour is the artificial initiation of uterine contraction by various means such as medical, surgical or mechanical with the aim of achieving vaginal delivery. Misoprostol, a prostaglandin E1 analogue is used popularly for the induction of labour in resource-limited health centres. The aim of the study was to find out the prevalence of induction of labour among pregnant women in the Department of Obstetrics and Gynaecology in a tertiary care centre.

**Methods:**

A descriptive cross-sectional study was conducted among pregnant women in a tertiary care centre from 3 February 2022 to 31 July 2022. Ethical approval was taken from the Institutional Review Committee. The women with a singleton pregnancy, reactive non-stress test, and adequate pelvis were included. Women with malpresentation, previous cesarean section, placenta previa, and cephalopelvic disproportion were excluded. Convenience sampling method was used. The point estimate was calculated at a 95% Confidence Interval.

**Results:**

Among 1355 pregnant women, the prevalence of induction of labour was found to be 135 (9.96%) (8.37-11.55, 95% Confidence Interval).

**Conclusions:**

The prevalence of induction of labour among pregnant women was found to be similar to other studies done in similar settings.

## INTRODUCTION

Induction of labour (IOL) is the artificial initiation of uterine contraction by various means such as medical, surgical or mechanical to achieve vaginal delivery. Misoprostol, a prostaglandin E1 analogue is used popularly for IOL in resource-limited health centres. It is a common obstetrics intervention performed when the continuation of pregnancy is not desired.^1^

The IOL rate varies from different parts of the world. In the United States, the rate has risen steadily from 9.6-27.1 of all birth from 1990-2018.^2^ The rate of induction in Nepal was reported between 8.9% to 10.5% by different authors.^3,4^ Induction is indicated when benefits outweigh the harms more to mother and fetus than induction itself.^5^ IOL may sometimes have adverse effects on the health of the mother and

fetus if a selection of cases and their monitoring is not done properly.

The aim of the study was to find out the prevalence of induction of labour among pregnant women in the Department of Obstetrics and Gynaecology in a tertiary care centre.

## METHODS

This descriptive cross-sectional study was conducted among pregnant women in the Department of Obstetrics and Gynaecology in Birat Medical College Teaching Hospital, Biratnagar, Morang, Nepal, from 3 February 2022 to 31 July 2022. Ethical approval was taken from the Institutional Review Committee (Reference number: IRC-PA-193/2078-79). The women with a singleton pregnancy, reactive non-stress test, and adequate pelvis were included. Women with malpresentation, previous cesarean section, placenta previa, and cephalopelvic disproportion were excluded. Convenience sampling method was used. The sample size was calculated using the following formula:


$$\begin{array}{ll} \text{n} & ={\text{Z}}^{2}\times \frac{\text{p}\times \text{q}}{{\text{e}}^{\text{2}}}\\ & =1.96^{2}\times \frac{0.09\times 0.09}{0.02^{2}}\\ & =787 \end{array}$$

Where,

n = minimum required sample size
Z = 1.96 at 95% of Confidence Interval (CI)
p = prevalence of induction of labour taken from previous study as, 9%^6^
q = 1-p
e = margin of error, 2%

The minimum required sample size was 787. However, the final sample size taken was 1355.

The patients were admitted to the antenatal ward one day prior to induction. All the prerequisites for IOL were checked.^6^ A reliable estimation of gestational age, presentation and foetal weight was done. Maternal pulse, blood pressure, temperature, respiratory rate and findings on abdominal palpation were recorded. The evaluation of baseline fetal heart rate pattern was done by auscultation/electronic foetal monitoring. Maternal pelvis assessment and clinical evaluation for possible cephalopelvic or feto-pelvic disproportion was done. Cervical status was assessed using modified Bishop scoring system to predict the likelihood of success and to select an appropriate method of induction of labour.^7^ Informed and written consent was taken. The possible risks associated with IOL were well explained. The patients were induced at 4 a.m. in the labour room with the first dose of misoprostol. Maternal and fetal monitoring was done properly. Maternal vitals and contractions were taken at regular intervals. Fetal heart rate was monitored with a non-stress test and intermittent auscultation. Patients with live fetuses were induced with a maximum of 3 doses of misoprostol whereas, in case of fetal death, induction was done with a maximum of 5 doses. The contraction was checked every 30 minutes. Maternal outcomes were assessed in terms of mode of delivery and complications.^1,4^

Data were entered in Microsoft Excel 2016 and analysed using IBM SPSS Statistics version 21.0. The point estimate was calculated at a 95% CI.

## RESULTS

Among 1355 pregnant women, the prevalence of induction of labour was found to be 135 (9.96%) (8.37-11.55, 95% CI). Majority of patients, 102 (75.55%) belonged to the age group of 20-30 years (Table 1).

**Table 1:** Age-wise distribution of the patients (n= 135).

Age (years)	n (%)
<20	9 (6.67)
20-30	102 (75.55)
31-40	24 (17.78)

Among 135 women, 78 (57.78%) were primigravida and 57 (42.22%) were multigravida. Postdated pregnancy was the commonest 73 (54.07%) followed by premature rupture of membrane (PROM) in 18 (13.33%) and hypertension in 9 (6.67%) (Table 2).

**Table 2:** Indications of induction of labour (n= 135).

Variables	n (%
Post dated	73 (54.07)
Premature rupture of membranes (PROM)	18 (13.33)
Hypertensive disorder of pregnancy	9 (6.67)
Rh negative pregnancy	4 (2.96)
Obstetrics cholestasis	7 (5.18)
Gestational diabetes mellitus (GDM)	3 (2.22)
Oligohydramnios	7 (5.18)
Decreases fetal movement	5 (3.70)
Intrauterine growth restriction (IUGR)	3 (2.22)
Prolonged latent stage of labour (LSOL)	4 (2.96)
Intrauterine fetal death (IUFD)	2 (1.48)

**Figure 1:**
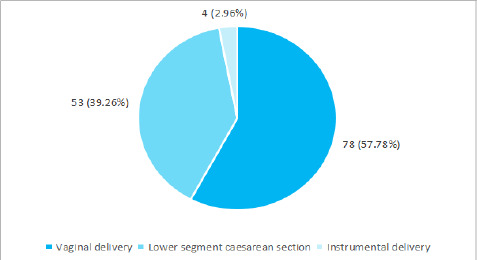
Mode of delivery (n= 135)

Among 135 patients, 78 (57.78%) were delivered via vaginal delivery, 53 (39.26%) delivered by lower section caesarean section (LSCS) and 4 (2.96%) by instrumental delivery (Figure 1).

Maternal complications were noticed only in 22 (16.30%) and the majority 113 (83.70%) of patients had no known complication. However, no maternal mortality was reported.

## DISCUSSION

Out of 1355 pregnant women, the prevalence of induction of labour was found to be 135 (9.96%). Induction of labour is a common intervention practised in modern obstetrics. It is indicated when pregnancy continuation imposes a risk to the health of the mother and fetus. Induction of labour has both positive and negative impacts on the mother and fetus. The prevalence of induction of labour in our study is within range compared to other institutions and other studies in Nepal.^3,4^ The prevalence in our study was similar to a study done in north Ethiopia (9%).^6^ In the present study a maximum number of patients were in the age group of 20-30 years 102 (75.56%). The most common indication for induction was postdated pregnancy 73 (54.07%) which is comparable to a study (51.28%).^8^

Likewise, regarding mode of delivery, in the present study 78 (57.78%) were delivered via vaginal delivery, 53 (39.26%) delivered by lower section caesarean section and 4 (2.96%) by instrumental delivery. A similar study reported vaginal delivery in 64.9%, LSCS in 25.8% and instrumental delivery in 9.30%.^9^ While comparing with another study, the vaginal delivery is lower in our study, however, the LSCS is slightly higher and the instrumental delivery is also lesser in our studies. The reason for variation could be the different times of presentation of pregnant women in different settings and their health status.

The most common indication for LSCS in our study was failed induction in 47.09% followed by fetal distress in 39.06%. Similarly, results were reported in a study in which the most common induction for LSCS was failed induction in 44% of cases followed by fetal distress in 29%.^10^ In another study, failed induction of labour was seen in 20.5%.^10^ In another study, 90% had a successful vaginal delivery.^12^

The limitations of the study are that the study is done in a single medical institution, so this research cannot be generalized to all the other places. Also since this is a descriptive cross-sectional study, different analytical parameters could not be measured.

## CONCLUSIONS

The prevalence of induction of labour among pregnant women was found to be similar to other studies done in similar settings.
